# Hass Avocado (*Persea americana* Mill) Peel Extract Reveals Antimicrobial and Antioxidant Properties against *Verticillium theobromae*, *Colletotrichum musae*, and *Aspergillus niger* Pathogens Affecting *Musa acuminata* Colla Species, in Ecuador

**DOI:** 10.3390/microorganisms12091929

**Published:** 2024-09-23

**Authors:** Caterine Donoso, Mihai A. Raluca, Stephanie Chávez-Jinez, Edwin Vera

**Affiliations:** 1 Departamento de Ciencias de Alimentos y Biotecnología, Facultad de Ingeniería Química y Agroindustria, Escuela Politécnica Nacional EPN, Quito 170143, Ecuador; caterine.donoso@epn.edu.ec (C.D.); edwin.vera@epn.edu.ec (E.V.); 2 Army Scientific and Technological Research Center—CICTE, Department of Life Science and Agriculture, Universidad de Las Fuerzas Armadas—ESPE, Av. General Ruminahui s/n y, Sangolqui 171103, Ecuador; sschavez3@espe.edu.ec

**Keywords:** Hass avocado (*Persea americana* Mill) peel, *Verticillium theobromae*, *Colletotrichum musae*, *Aspergillus niger*, *Musa acuminata* Colla, phenolic compounds, antioxidant activity, agroindustry residues

## Abstract

The utilization of agroindustrial residues, such as avocado peel, as a source of bioactive compounds with antioxidant properties has garnered significant attention. In this study, we investigated the antioxidant potential using the DPPH (2,2-diphenyl-1-picrylhydrazyl) and ORAC (oxygen radical absorbance capacity) methods, along with the antimicrobial activity of phenolic compounds extracted from Hass avocado peel. These soluble polyphenols were quantified and identified using high-performance liquid chromatography (HPLC). The research focused on their effects against three fungal pathogens, *Verticillium theobromae*, *Colletotrichum musae*, and *Aspergillus niger*, which significantly impact banana crops, an essential agricultural commodity in Ecuador. The results have revealed that the application of 80% ethanol as an organic solvent led to increased soluble polyphenol content compared to 96% ethanol. Extraction time significantly influenced the phenolic content, with the highest values obtained at 90 min. Interestingly, despite substantial mycelial growth observed across all extract concentrations, the antifungal effect varied among the pathogens. Specifically, *V. theobromae* exhibited the highest sensitivity, while *C. musae* and *A. niger* were less affected. These results underscore the importance of considering both antioxidant and antimicrobial properties when evaluating natural extracts for potential applications in plant disease management.

## 1. Introduction

Humanity is progressively working towards a comprehensive strategy to transition from a linear to a circular economy, which includes various initiatives aimed at minimizing the vast amounts of waste produced each year. Food waste represents a renewable resource that can be harnessed and transformed into valuable products, which not only helps decrease the amount of waste sent to landfills but also enhances the market potential for new sustainable goods [1]. One significant area of focus is avocado waste, especially for the high commercial utilization of avocado that generates massive amounts of avocado bio-wastes, including peel and seed by-products.

Avocado seeds are proved to contain a rich array of bioactive compounds, including polyphenols, triterpenoids, acetogenins, and fatty acids that contribute to various health benefits, such as antihypertensive, antimicrobial, antioxidant, larvicidal, and hypolipidemic effects. Notably, avocado seed extracts have shown promise in managing diabetes by reducing blood glucose and in Alzheimer’s disease management, supported by in vitro studies demonstrating antioxidant and anti-cholinesterase activities [2].

Also, avocado peel, often considered waste after industrial avocado processing, holds hidden treasures. Rich in natural compounds like triterpenes, phytosterols, and polyphenols, avocado peel can constitute up to 13% of the avocado’s weight. Unfortunately, these tons of peel by-products are typically discarded or used only as animal feed.

Hass avocado (*Persea americana* Mill) peel has generated attention due to its versatile potential, containing over 35 phytochemical compounds with diverse beneficial applications in cosmetics, food, and pharmaceuticals, acting as anti-inflammatory, antioxidant, and antimicrobial agents [3]. These compounds include phenolic acids, condensed tannins, carotenoids, flavonoids, flavanols, and hydroxybenzoic and hydroxycinnamic acids. Notably, flavonoids derived from amino acids (phenylalanine and tyrosine) are present, such as catechin, epicatechin, procyanidins, quercetin, rutin, kaempferol, persin, and 3-o-caffeoxylquinic acid [4]. The presence of hydroxyl groups (OH) attached to the aromatic rings in these molecules enables the reduction of oxidative stress by capturing free radicals.

However, recent research highlights the remarkable properties of avocado peel. Beyond its known anti-hypertensive and hypolipidemic properties, avocado peel also exhibits fungicidal and larvicidal effects [5]. Importantly, studies demonstrate that avocado peel extracts possess strong antibacterial activity against various bacteria, including *Escherichia coli*, *Pseudomonas aeruginosa*, *Enterococcus faecalis*, *Proteus vulgaris*, and *Staphylococcus aureus* [6].

Given Ecuador’s reliance on banana cultivation, harnessing avocado peel’s antimicrobial potential could offer a sustainable solution to combat fungal diseases affecting bananas.

In particular, *Musa acuminada* Colla is the primary banana species cultivated worldwide. India leads as the largest banana producer, followed by China, the Philippines, Ecuador, and Brazil [7]. According to the Food and Agriculture Organization (FAO) of the United Nations, banana cultivation significantly contributes to Ecuador’s socio-economic development. It generates extensive employment opportunities for over a million families, involving approximately 2.5 million individuals, both native and non-native to the region, constituting almost 17% of Ecuador’s total population [8]. Notably, banana production is predominantly in the hands of Ecuadorian communities and families, fostering a popular and solidarity-based economy, particularly in the provinces of El Oro (14%), Guayas (34%), and Los Rios (16%).

The trend toward maintaining a healthy lifestyle has been a key factor in increasing the demand for fruit consumption, resulting in revenues of USD 241 million [9]. As one of the most widely consumed and traded fruits globally, bananas face challenges due to their susceptibility to various diseases. These include cigar tip, anthracnose, and rot caused by the pathogens *Verticillium theobromae*, *Colletotrichum musae*, and *Aspergillus niger*, respectively. These pathologies affect the vascular system and tissues due to fungal mycotoxins, hindering nutrient absorption. Consequently, visual lesions such as dark spots may appear, leading to fruit decomposition and significantly impacting both quality and food safety [10]. These issues result in losses of 20% to 25% of the total production [11], with specific losses of 15% to 50% attributed to *Verticillium theobromae*, as reported in 2005 [12].

Economic losses in banana cultivation extend beyond reduced income due to decreased fruit production, including inferior quality and additional costs arising from fungicide application, disease management, and the potential necessity of replanting the affected area. Consequently, the global demand for bananas, coupled with the imperative to minimize chemical pesticide use for pest control, underscore the urgency of developing sustainable and effective alternatives for managing fungal diseases in banana crops.

In this context, the avocado, also known as “green gold”, enjoys high global consumption, with exports totaling 1300 tons. Consequently, the substantial demand for avocados in various agro-industrial activities generates waste, including Hass avocado (*Persea americana* Mill) peels.

These peels have generated attention due to their versatile potential, containing over 35 phytochemical compounds with diverse beneficial applications in cosmetics, food, and pharmaceuticals.

The objective of this research is to take advantage of an agroindustrial waste product, Hass avocado peel, by extracting phenolic compounds, such as chlorogenic acid, catechin, and epicatechin, with antimicrobial activity against pathogens affecting one of the most exported crops in Ecuador. By repurposing this overlooked resource, we may contribute to healthier banana crops and a more resilient agricultural sector.

## 2. Materials and Methods

### 2.1. Reagents

A Milli-Q water purification system was used for ultrapure water. 1,1-diphenyl-2-picrylhydrazyl (DPPH), 6-hydroxy-2,5,7,8-tetramethylchroman-2-carboxylic acid (TROLOX), 2,2′-Azobis(2-methylpropionamidine) dihydrochloride (AAPH), 98% from Acros organics (Geel, Belgium), Folin-Ciocalteu’s, sodium hypochlorite, n- hexane 95% ACS from Fermont, reagents of analytical grade like ethanol and methanol, Gallic acid (97.5–102.5%) from Sigma-life science, di-Potassium hydrogen phosphate (anhydrous) and Potassium di-Hydrogen Phosphate for analysis, ACS from AppliChem Panreac (Darmstadt, Germany), Fluorescein sodium salt from Sigma-Aldrich (St. Louis, MO, USA), sodium carbonate from JT Baker (Phillipsburg, NJ, USA). Acetonitrile from Supelco (Bellefonte, PA, USA), Ethanol and methanol of HPLC grade, pure nitrogen (99.998%).

### 2.2. Obtention of Plant Material

Avocados of the Hass variety (*Persea americana* Mill) were obtained from the province of Pichincha, located 45 km north of the city of Quito, with GPS coordinates −0.12981, −78.48202. The avocados were subsequently transferred to the Department of Food Sciences and Biotechnology (DECAB) at the Escuela Politécnica Nacional.

### 2.3. Sample Preparation

A sodium hypochlorite solution (50%, *v*/*v*) was prepared, and the avocados were immersed in it for subsequent disinfection for 10 min. The avocado peels were then removed and stored in Ziploc bags and placed in an ultrafreezer at −82 °C for 2 days. The lyophilizer equipment was conditioned to −60 °C for 72 h, and the avocado peels were introduced. After this process, the peels were triturated using a food processor, and the resulting fine powder was sieved and stored in hermetically sealed bags with an aluminum coating.

For the defatting stage of the obtained fine powder, hexane was used in a ratio of 1:20 (*m*/*v*). The plant material and solvent were combined in Falcon tubes and agitated for 2 min, followed by 10 min of centrifugation.

### 2.4. Avocado Peel Extraction

A solution was prepared with a ratio of 1: 20 (*m*/*v*), following the methodology proposed by [13,14], with experimental adjustments based on preliminary studies by [15]. Ultrasound-assisted extraction was performed using two extraction times (90 min/120 min) and two ethanol concentrations as the solvent (80%/96%). The ultrasound conditions were set at a frequency of 60 Hz and a power of 120 W, maintaining a temperature of 25 °C in a dark environment. The resulting extract was then centrifuged and stored in amber vials at −20 °C for subsequent analysis.

### 2.5. Determination of the Total Soluble Polyphenol Content

The total phenolic content of the avocado peel extracts was determined using the Folin–Ciocalteu assay of the European Commission [16] with few modifications. Aliquots of 100 μL from the four avocado peel extracts were diluted (1: 50, *v*/*v*) with Type II water. Next, 20 μL of the extract was mixed with 100 μL of the Folin-Ciocalteu reagent (1:4, *v*/*v*). The mixture was agitated for 60 s in a flat-bottomed 60-well plate. Subsequently, the mixture was allowed to rest for 240 s, followed by the addition of 75 μL of a 100 g/L Na_2_CO_3_ solution. After 2 h at room temperature (25 °C), the absorbance was measured using a UV-VIS spectrophotometer (Bio Tek Instruments, Winooski, VT, USA) at 750 nm.

A gallic acid (GA) calibration curve was obtained using standard solutions within the range of 10–200 mg/L. The results are expressed as mg GA equivalent/g of palm leaves (mg GAE/g DW). The obtained equation was expressed as y = 0.0083x + 0.0034, and the correlation coefficient of the calibration curve was 0.9995.

### 2.6. HPLC Analysis of Avocado Peel Extract

The High-Performance Liquid Chromatography (HPLC) assay was conducted under specific conditions. As the stationary phase, an analytical C18 column was employed, which was conditioned at 40 °C using the Waters e2695 Xbridge equipment (Waters Corporation, Milford, MA, USA. The analysis was performed on the sample that exhibited the highest content of phenolic compounds and the greatest antioxidant capacity. For the mobile phase, Type I water for HPLC acidified with 0.1% formic acid and HPLC-grade methanol (30:70 *v*/*v*) was used, with a 1 mL/min flow rate through the column.

### 2.7. Antioxidant Activity

#### DPPH Radical Scavenging Assay

The DPPH (2,2-diphenyl-1-picrylhydrazyl) activity was determined using the method described by [16], with several modifications. A microplate was prepared, where 20 µL of diluted sample was placed, followed by 180 µL of the DPPH stock solution at a concentration of 150 µM. The same procedure was performed with various dilutions of Trolox for the calibration curve. The microplate was then placed in a dark environment and read using a microplate reader. The absorbance was measured at 515 nm over 40 min. The DPPH scavenging ability was expressed as a percentage in terms of µM equivalent Trolox per liter of solution and calculated as the difference between the absorbances.

$$DPPH\;inhibition\;(\%)=\left[1-\frac{A_{sample}-A_{blank}}{A_{control}-A_{blank}}\right]\times 100$$

Afterward, we calculated a curve of % DPPH bleaching activity versus concentration. The calibration curve was determined using TROLOX standard solutions ranging from 61.2 to 0.612 µM, obtaining an equation of y=0.1341x−1.3209 with a correlation factor of $R^{2}=0.9998$; *p* = 0.0363.

### 2.8. ORAC Oxygen Radical Absorption Capacity Assay

The ORAC (oxygen radical absorbance capacity) activity was determined using the method described by [17]. A fluorescein solution was prepared at a concentration of 140 mM, and an AAPH (2,2′-Azobis(2-methylpropionamidine) dihydrochloride) solution was prepared at 0.40 mM. The Trolox calibration curve was established using solutions ranging from 25 to 800 µM.

In a flat-bottomed black microplate, 20 µL of the avocado peel extract was placed, followed by the addition of 120 µL of fluorescein. The microplate was then placed in a microplate reader at 37 °C for 15 min. The same procedure was performed with Trolox solutions as standards and a buffer solution as the blank. Subsequently, 60 µL of AAPH was added, and absorbance was measured at 485 nm (for wavelength) and 520 nm (for emission) over 80 min.

The resulting curve represented the fluorescence loss over time (fluorescence vs. time), and the area under the curve (AUC) was determined for the sample, blank, and standards. Additionally, the differences between the AUC of the blank and the sample/standard were calculated using the following formula,

$$ABC=1+\sum_{i=1}^{i=80}f_{i}/f_{0}$$

Obtaining an equation of y=0.0842x+1.6816 with a correlation factor of $R^{2}=0.997;p$ =0.0130.

### 2.9. Antifungal Activity Assay

#### 2.9.1. Inoculum Preparation

A 0.01% Tween solution was prepared and used for fungal scraping onto Petri dishes. The resulting suspension was transferred to test tubes, vortexed for 1 min, and then 25 µL was placed in a Neubauer chamber for conidial counting under a microscope at 40× magnification. Based on the calculated actual concentration, the volume required to achieve a concentration of (10^6^) conidia/mL was determined and added to 20 mL of Tween 80 solution.

#### 2.9.2. Dilution Method in PDA Patato Dextrose Agar

The dilution method in PDA was employed, including a control group treated with a commercial fungicide [18] and an absolute control using the PDA medium alone. Different concentrations were prepared (ranging from 200 to 1000 ppm and 1000 to 2200 ppm) depending on the specific fungus. To determine the minimum concentration of extract capable of inhibiting mycelial growth, concentrations from 200 to 1000 ppm were studied in *Verticillium theobromae* and *Colletotrichum musae*. In the case of *Aspergillus niger*, the same concentrations had no inhibitory effect in previous assays; for this reason, concentrations higher than 1000 ppm were studied where a percentage of inhibition was already evident.

For the concentration of the ethanolic extracts, the Glas Col evaporator was used at 21 °C, bubbling extra pure nitrogen (99.998%) at a pressure of 166 bar to evaporate the ethanol and avoid its interference in the growth inhibition study of the phytopathogens.

The extract without ethanol was mixed with PDA using a magnetic stirrer, and 20 mL of the mixture was poured into each Petri dish. After solidification, a portion of the fungus was placed in the center of each Petri dish, sealed with parafilm, and incubated in darkness at 25 °C until the mycelium reached the edge of the plate in the control treatments, following the procedure reported by [18]. Four replicates were performed for each treatment, and radial mycelial growth was measured (in mm). The percentage of growth inhibition (PI) for each propyl disulfide concentration was calculated using the following formula:

$$\%\;inhibition=[\frac{\left(mm\;growth\;control\right)-\left(mm\;growth\;treatment\;.\right)}{mm\;growth\;control}]\times 1$$

### 2.10. Statistical Analysis

In the experiment, three replicates were used for each treatment, and the results are expressed as means ± standard deviation (SD). To test differences, an analysis of variance (ANOVA) was applied. These tests assessed differences between categories related to extraction time and solvent concentration for both total polyphenol content and antioxidant activity. The Shapiro-Wilk test was used to verify homoscedasticity and normality.

## 3. Results

### 3.1. Total Soluble Polyphenol Content

The highest concentration of soluble polyphenols among the four extraction treatments was achieved under the conditions of 80% ethanol and 1 h 30 min of extraction, followed by 96% ethanol at the same duration. Conversely, the lowest yields were observed under the conditions of 80% ethanol for 2 h and 96% ethanol for 2 h, as recorded in Table 1.

**Figure 1:**
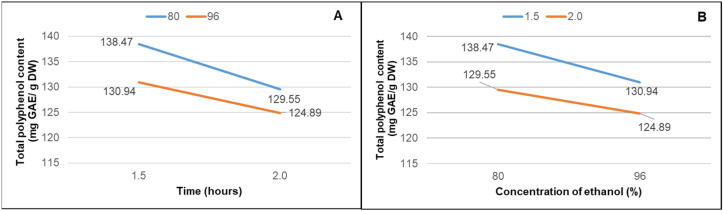
The interaction effects of extraction time and ethanol concentration on the total phenolic content (TPC) (**A**) Interaction of extraction time with TPC: indicate a null interaction effect between extraction time and TPC. Notably, there were no highly significant differences observed between different extraction times. (**B**). Interaction of ethanol concentration with TPC. Similarly, the interaction effect between ethanol concentration and TPC was also null. No highly significant differences were observed based on varying ethanol concentrations.

Duncan’s test of analysis of variance (ANOVA) (*p* < 0.0001) indicated significant differences between extraction time and ethanol concentration. The adjusted R-squared value of 0.74 suggests that approximately 74% of the variation in polyphenol concentration across different extracts is influenced by both time and ethanol concentration. Figure 1 illustrates the interaction between the study variables (extraction time and ethanol concentration).

### 3.2. HPLC Analysis

**Figure 2:**
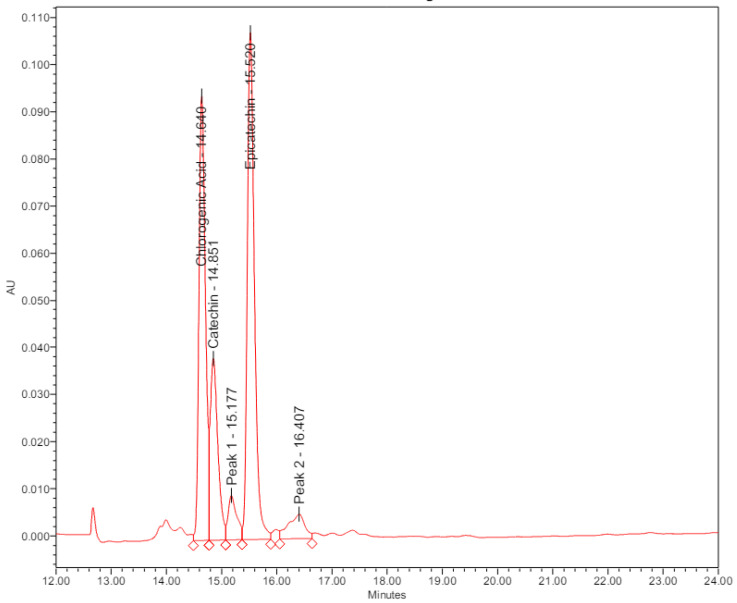
HPLC analysis of TI extract with the following compounds identified chlorogenic Acid Catechin Epicatechin, with retention time in min and maximum relative abundance.

As indicated in Table 2 and Figure 2, the three compounds with the highest relative peak areas, along with their respective percentages, were as follows: epicatechin (41.74%), chlorogenic acid (32.98%), and catechin (16.17%), and two unidentified structural isomers with percentages of (4.45%) and (4.66%).

### 3.3. Antioxidant Activity

The antioxidant properties of the four extraction treatments from Hass avocado peel were evaluated using two hydrogen atom transfer/based methods. Notably, minimal significant differences were observed among the treatments, as reported in Table 3.

**Figure 3:**
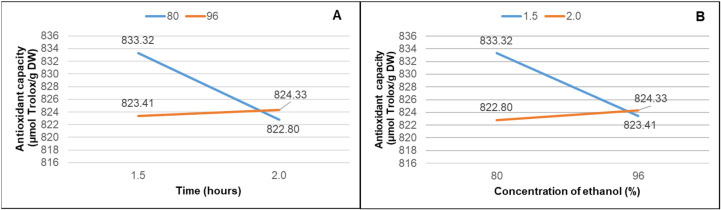
The effect of the interaction between extraction time and DPPH antioxidant capacity (**A**) and the interaction between ethanol concentration and DPPH antioxidant capacity (**B**).

By evaluating the structure–activity relationship, this study revealed the low OH substitution at the 3′ position of the pyrogallol concerning their non-3′-OH counterparts such as epicatechin, which contributes to a higher ORAC value for the DPPH method. Overall, the results of this study allowed us to hypothesize that at 80% and 96% ethanol–water concentrations, the formation of reactive oxygen species is not promoted. In Figure 3, an inverse interaction is evident, where the effect of ethanol percentage and time is nonlinear. Additionally, the relationship between them is not directly proportional to the obtained antioxidant capacity. This type of interaction suggests that the effect of ethanol percentage is not consistent across all levels of the time variable.

**Figure 4:**
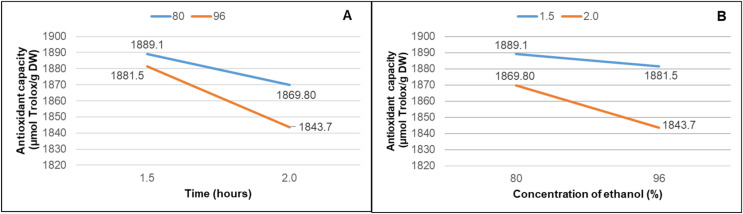
The effect of the interaction between extraction time and ORAC antioxidant capacity (**A**) and the interaction between ethanol concentration and ORAC antioxidant capacity (**B**).

Additionally, as shown in Figure 4, there is a null interaction effect between extraction time and ethanol concentration concerning antioxidant capacity. Interestingly, the highest antioxidant capacity was achieved when employing the shortest extraction time (1 h 30), regardless of the solvent concentration used.

### 3.4. Evaluation of the Antifungal Activity

In the agar dilution assay, it was observed that the percentage of growth inhibition of fungal pathogens was significantly higher for *Verticillum theobromae*, with an inhibition of 61.59% at a concentration of 1000 ppm, followed by *Colletrichum musae* with 35.67%. The lowest inhibition percentage was observed for *Aspergillus niger* at a concentration of 2200 ppm, with a value of 27.59 (Table 4 and Table 5).

**Figure 5:**
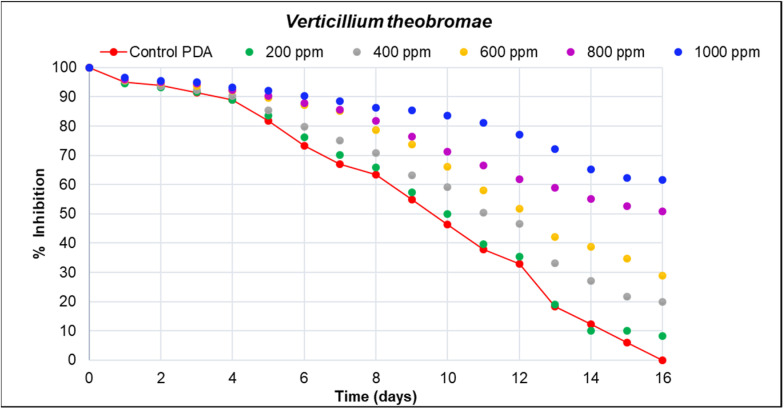
Effect of the hydroethanolic extract in the dilution assay on the growth of *V. theobromae*. The reported inhibition is the average of four replicates for each proposed concentration (200 to 1000 ppm). The red trend represents the control (PDA), and differences are depicted using a scatter plot. Notably, at 1000 ppm, the highest fungal inhibition and the least mycelial growth were observed.

**Figure 6:**
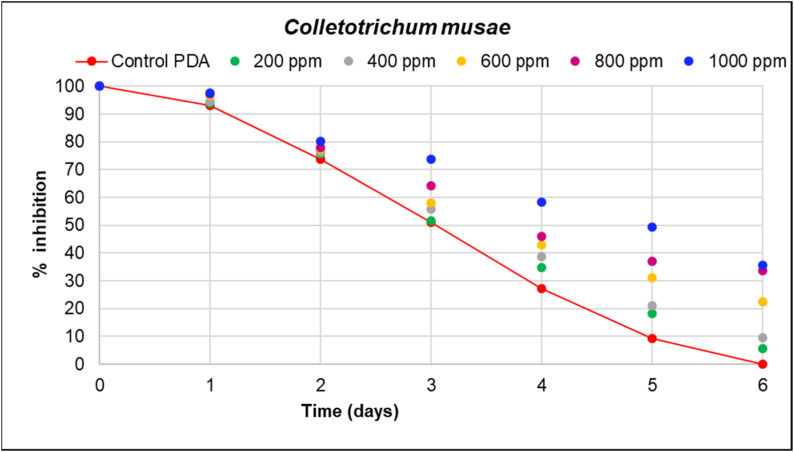
The effect of the hydroethanolic extract in the dilution assay on *C. musae* growth. The inhibition reported represents the average of four replicates for each proposed concentration (200 to 1000 ppm). The red trend represents the control (PDA). Notably, at 1000 ppm, the highest fungal inhibition and the least mycelial growth were observed.

**Figure 7:**
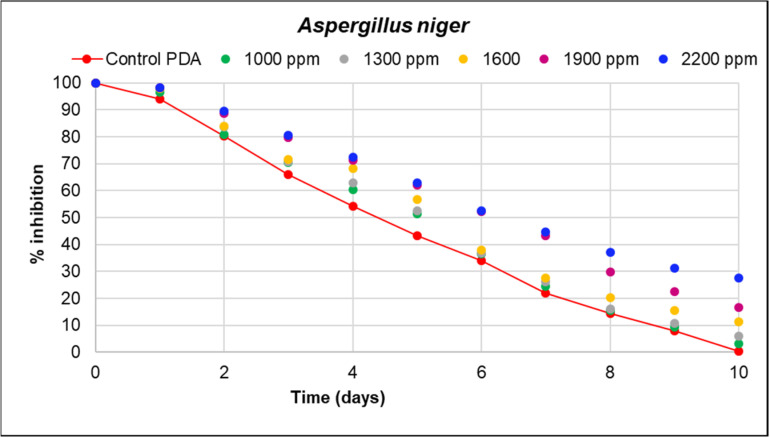
The effect of the hydroethanolic extract on *A. niger* growth. The reported inhibition represents the average of four replicates for each proposed concentration (1000 to 2200 ppm). The red trend represents the control (PDA). At 2200 ppm, the highest percentage of fungal inhibition and the least mycelial growth were observed.

In Figure 5, Figure 6 and Figure 7, the inhibition curves of *Verticillium theobromae*, *Colletotrichum musae*, and *Aspergillus niger* are evident. These curves represent the response of fungal growth inhibition to different concentrations of hydroethanolic avocado peel extract compared to a control (PDA). The maximum inhibition values were 61.59 mm, 35.67 mm, and 27.59 mm, respectively, corresponding to the highest concentration for each fungal pathogen. Significant differences were observed among the five treatments, as determined by Duncan’s test (*R*^2^ = 1.00). This indicates that 100% of the inhibition percentage is attributed to the extract concentration and the total time required for fungal growth. Refer to Table 3 and Table 4 for further details. Furthermore, the reported inhibition percentage increased with higher extract concentrations, resulting in smaller diameters for *V. theobromae*, *C. musae*, and *A. niger*.

**Figure 8:**
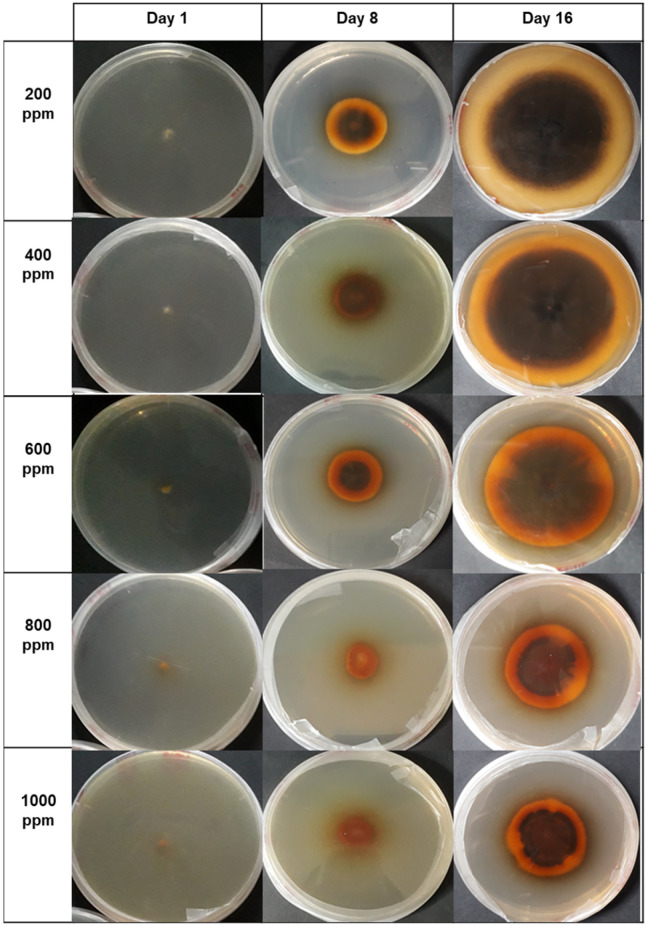
The inhibitory effect of polyphenols (catechin, epicatechin, chlorogenic acid, and isomers) on the growth of *V. theobromae* mycelium is depicted in the graph. From right to left, the days 1, 8, and the final day of fungal growth in the incubator are shown. Vertically, the concentrations applied in each Petri dish (ranging from 200 to 1000 ppm) are represented. The control group used the medium without extract.

**Figure 9:**
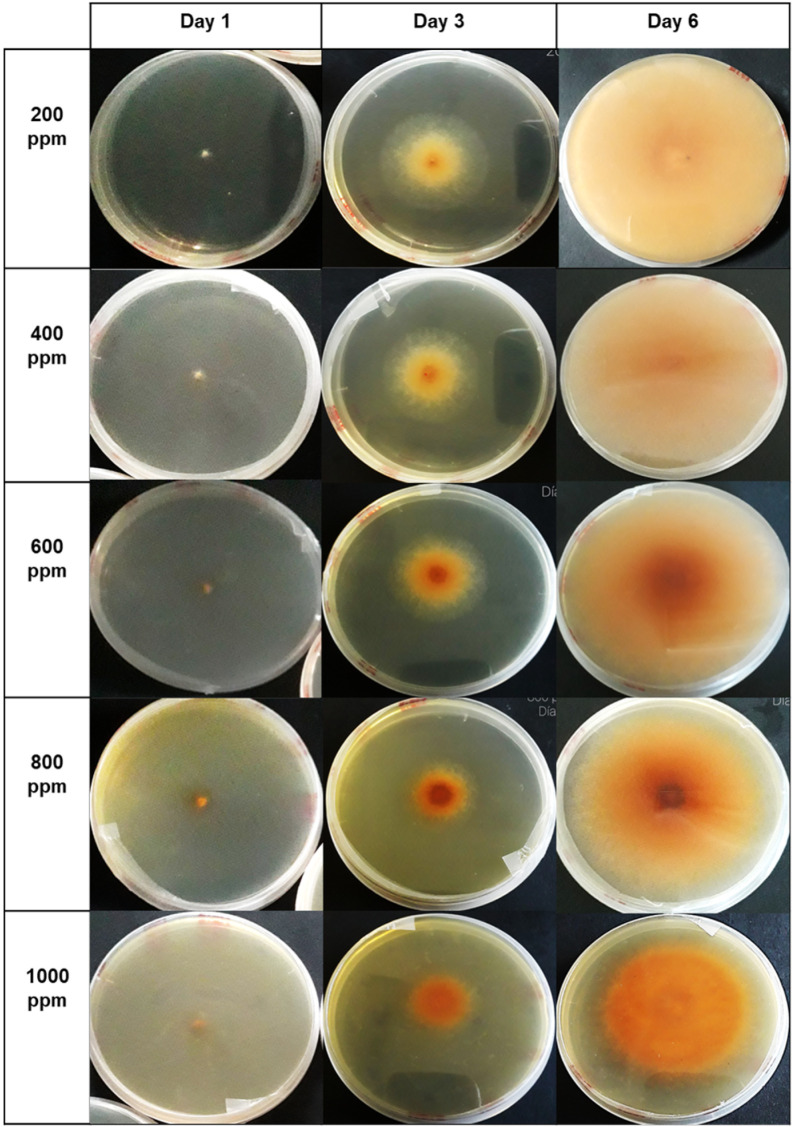
The inhibitory effect of polyphenols (catechin, epicatechin, chlorogenic acid, and isomers) on the growth of *C. musae* mycelium is depicted in the graph. From right to left, the days 1, 3, and the final day of fungal growth in the incubator are shown. Vertically, the concentrations applied in each Petri dish (ranging from 200 to 1000 ppm) are represented. The control group used the medium without extract.

**Figure 10:**
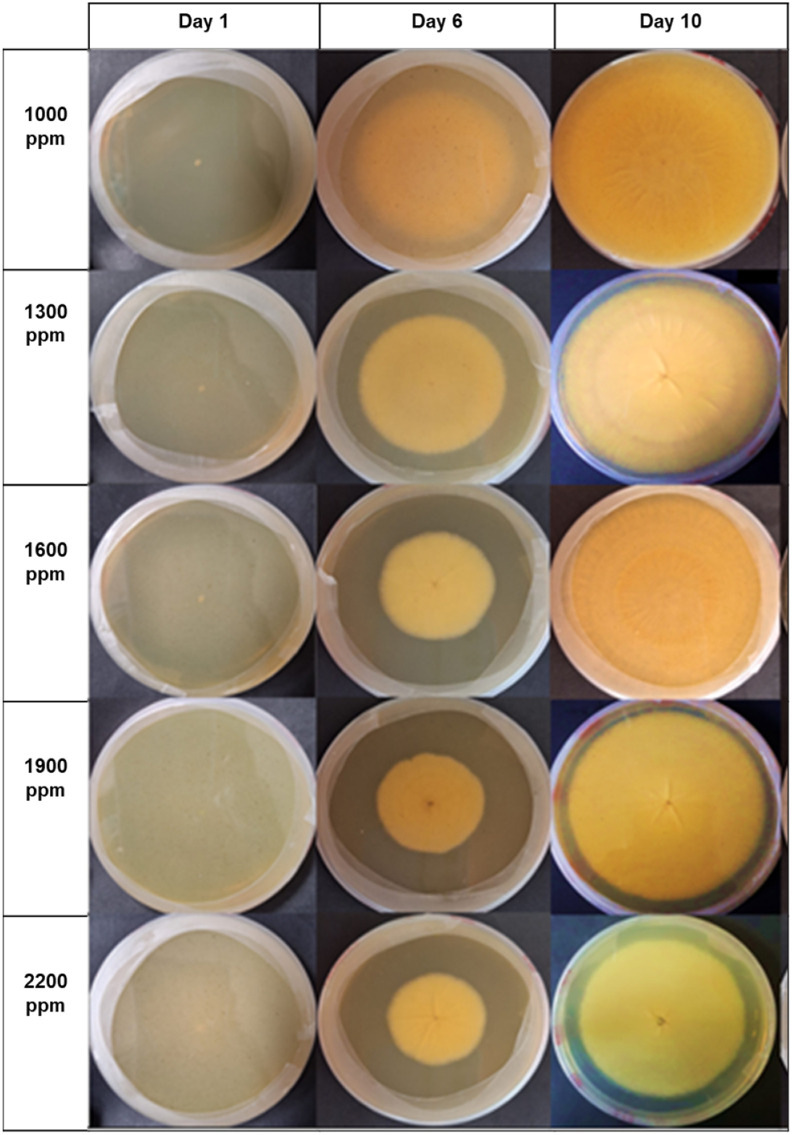
Inhibitory effect of polyphenols (catechin, epicatechin, chlorogenic acid, and isomers) on the mycelial growth of *A. niger*. In the graph, from right to left, we observe the mycelial growth of the fungus on days 1, 5, and the final day in the incubator. The corresponding concentration applied in each Petri dish (ranging from 200 to 1000 ppm) is depicted vertically. The control group used the medium without extract.

According to the organic compounds identified in the HPLC assay—specifically chlorogenic acid, catechin, and epicatechin—they were responsible for the changes observed in fungal growth. This is evident in Figure 8, Figure 9 and Figure 10. Notably, the presence of different concentrations of the hydroethanolic avocado peel extract led to color changes in the PDA medium, as well as variations in the tonalities associated with each fungus.

Regarding the assay with *Verticillium theobromae*, it is noteworthy that at concentrations ranging from 200 to 600 ppm, the fungal mycelium remains relatively intact, retaining its cotton-like structure. However, at higher concentrations—specifically from 800 to 1000 ppm—a decrease in the cotton-like mycelial structure is observed, resulting in a visually flatter appearance.

Visual changes observed in *Colletotrichum musae* occurred starting from the third day of incubation. These changes were less intense compared to the control plate. Specifically, the color of the fungus shifted from pink to beige. At higher concentrations (800 ppm to 1000 ppm) of the avocado peel extract, a reduction in hyphal growth within the fungal mycelium was evident. Additionally, the texture appeared less cotton-like and less compact, with a notable absence of spores. This contrasted with the growth of *Colletotrichum musae* on the control plate without the extract.

During the 10-day incubation period, visual changes were observed in *Aspergillus niger*. Starting from the second day of incubation, fungal growth increased significantly, resulting in the formation of elongated structures (*conidiophores*). Interestingly, until the sixth day of growth, the fungus did not produce any spores. On the sixth day, the fungus retained a whitish-to-cream color, and a reduced number of round, black spores were visible at the center of the fungal colony. Surrounding the spore colony, a loosely compacted cotton-like structure was evident, especially at concentrations ranging from 1000 to 1300 ppm.

Over the observation days, gradual mycelial production occurred, accompanied by a reduced number of spores and conidiophores. This effect was more pronounced at higher concentrations, specifically between 1600 and 2200 ppm. By the tenth day, significant fungal growth was evident across all five concentrations, with the most prominent inhibition observed at 2200 ppm.

## 4. Discussion

Agroindustrial residues such as avocado peel are considered a source of bioactive compounds with antioxidant properties. These compounds help reduce and prevent food oxidation by absorbing free radicals and inhibiting oxidative enzymes [19]. Studies using HPLC have identified three phenolic compounds with high antioxidant capacity: catechin, epicatechin, and chlorogenic acid, along with two unidentified isomers. The reported values were 175 mg EAG/g dry sample for catechins and epicatechins and 42.9 mg EAG/g dry sample for chlorogenic acid derivatives [20].

We evaluated the extraction conditions (time and ethanol %) of avocado peel polyphenols using ultrasound-assisted extraction. The results highlighted the crucial role of extraction parameters in achieving considerable values. Specifically, using 80% aqueous ethanol as the solvent for 1 h and 30 min at 25 °C and 40 kHz yielded the highest phenolic content (138.47 mg EAG/g dry sample), followed closely by 96% ethanol (130.94 mg EAG/g dry sample).

A similar study by [21] reported a phenolic concentration of 77.85 mg EAG/g dry sample, which was lower than our findings. The discrepancy can be attributed to the extraction method employed (ultraturrax and maceration), demonstrating that ultrasound extraction yields a higher quantity of compounds due to acoustic cavitation. This phenomenon involves the formation and collapse of vacuum bubbles in the solvent, leading to violent disruption and increased diffusion of polyphenols into the extract [22].

In another study by [23], extraction was performed at 51 °C for 1 h and 5 min using 40% ethanol, resulting in a phenolic content of 125.19 mg EAG/g dry sample. The difference in extraction time likely explains the variation in results. In the study made by [24], extraction lasted 15 min at 25 °C and 40 kHz, resulting in a content of 63.5 mg EAG/g dry sample for Hass avocado peel and a value of 120.3 mg EAG/g dry sample for the Fuerte variety. These results were lower than those obtained in our study, likely due to differences in extraction time.

Exposing the plant matrix to the solvent for too short a time prevents the complete release and diffusion of compounds. Conversely, prolonged exposure (over 2 h) leads to degradation of functional groups due to ultrasonic cavitation. Therefore, the optimal extraction time of 1 h and 30 min maximized yield, benefiting from rapid acoustic cavitation that enhances polyphenol diffusion [25].

It is important to emphasize that the application of aqueous ethanol (alcohol/water) as an organic solvent at 80% increased the polyphenol concentration compared to pure 96% ethanol. This increase is attributed to enhanced solubility and isolation of polyphenols from the plant matrix into the solvent, as affirmed by [26].

The phenolic content at 1 h and 30 min (130.94 mg EAG/g dry sample) and 2 h (124.89 mg EAG/g dry sample) using 96% ethanol was lower than when using 80% ethanol (138.47 mg EAG/g dry sample and 128.69 mg/g, respectively). This difference can be attributed to the high affinity of hydroethanolic extracts for low-molecular-weight compounds such as chlorogenic acids and flavonoids (catechin, epicatechin) [27]. The hydrogen bonds present in the extract molecules become weaker when in contact with water, allowing for the breakdown of cellular structures and facilitating the release of these compounds. This phenomenon was observed in the study by [28], which reported a value of 144.7 mg EAG/g dry sample using 70% ethanol, higher than the value reported in this study.

In another investigation by [29], maceration was used with a non-organic solvent (hexane), resulting in a phenolic content of 26.33 mg EAG/g dry sample, which is lower than the values reported here. This limitation is due to the non-polar and highly selective nature of hexane toward lipids and oils, which restricts the extraction of polar compounds present in the avocado peel [30]. The application of hydroethanolic solvent with ultrasound waves facilitated the breakdown of particles with a size of 212 µm, resulting in increased surface area and efficient mass and energy transfer [31].

Using multiple methods to determine antioxidant capacity provides a broader understanding of the mechanism of action and relevant factors that may influence the obtained results [32].

The average highest antioxidant capacity reported in Table 3 of this study, based on three repetitions of the DPPH assay, corresponded to extract A1 (84.97%) with a concentration of 833.32 µmol Trolox/g dry sample, followed by extract B2 (84.04%). Previous research by [33] reported a value of 900.4 µmol Trolox/g dry sample, which closely aligns with the results obtained here. Additionally, they reported a value of 502.8 µmol Trolox/g dry sample using an aqueous avocado peel extract, which is lower due to the lower polar phenolic composition in that extract. In the study by [28], the DPPH scavenging ranged from 73.4% to 99%, consistent with the values reported in this study (ranging from 83% to 85%), and in agreement with the value of 85.2% obtained by [34]. These significant values are attributed to the effect of the high phenol content obtained using 80% ethanol and controlled ultrasound temperature, demonstrating increased electron transfer and DPPH radical reduction, resulting in a stable (non-reactive) form and a decrease in absorbance with a corresponding color change.

The ORAC assay, considered more sensitive to hydrophilic compounds due to hydrogen atom transfer, exhibited behavior similar to the DPPH assay. The highest antioxidant capacity was observed in extract A1 (1889.1 µmol Trolox/g dry sample), followed by extract B1 (1881.5 µmol Trolox/g dry sample). The most influential parameter was the extraction time (1 h and 30 min). These values correlate with the previously found total phenolic content, as phenolic structures provide hydrogen atoms [35].

Furthermore, ref. [33] reported a value of 12,541.2 µmol Trolox/g, which is 7 times higher than the value reported in our study. This discrepancy can be attributed to the quantity of polyphenols found by Trujillo, corresponding to 297.42 mg GAE (gallic acid equivalents) per gram of dried sample. This higher polyphenol content allows for greater absorption of peroxyl radicals (ROO) generated by the hydroethanolic avocado peel extract, resulting in the inhibition of fluorescence decay and a decrease in absorbance. In contrast, Ref. [26] applied an extraction using a mixture of acetone, water, and acetic acid for 5 min under ultrasonication. Their results yielded lower values compared to our study: 631.4 µmol Trolox/g for ORAC (oxygen radical absorbance capacity) and 189.8 µmol Trolox/g for DPPH (2,2-diphenyl-1-picrylhydrazyl). This finding confirms that using a shorter extraction time and less polar solvents (compared to ethanol) reduces the antioxidant capacity. The slower diffusion of compounds into the solvent necessitates a prudent extraction time, ultimately enhancing the efficacy of the obtained extracts.

Other research suggests by evaluating the structure-activity relationship that OH substitution at the 3′ position in pyrogallol moieties contributes to the lower ORAC value of epigallocatechin and epigallocatechin gallate compared with their non-3′-OH counterparts, such as epicatechin and epicatechin gallate, respectively. A lower TAC value in the ORAC assay compared with that in DPPH assays may pertain to a pro-oxidant effect by generating reactive oxygen species in an aqueous buffer, at a physiological pH [36]. These results differ from those obtained in this research because the extraction was carried out with ethanol concentrations between 80 and 96%.

It is essential to note that there are limited previous studies evaluating the antifungal activity of avocado peel extracts against banana fungal pathogens. Antifungal activity involves specific mechanisms that may not be directly related to antioxidant capacity. These mechanisms include interference with fungal cell wall synthesis, inhibition of DNA synthesis, or disruption of membrane function. Therefore, the purpose of this research was to explore the potential antifungal activity present in a hydroethanolic extract and compare it with extracts from various plant matrices. In the in vitro evaluation of antifungal activity, the extract (designated as A1) with the highest antioxidant capacity was tested against the pathogens *V. theobromae*, *C. musae*, and *A. niger*. The results indicated that at the proposed concentrations (200 ppm to 1000 ppm for *V. theobromae* and *C. musae*, and 1000 ppm to 2200 ppm for *A. niger*), the effect of extract A1 was fungistatic. This means that it reduced fungal growth, slowing down its development and inducing visual morphological changes. However, complete inhibition and cell death were not observed.

When the PDA medium came into contact with the hydroethanolic extract, a color change occurred—from translucent beige to translucent yellow. This color alteration can be attributed to the presence of compounds that may affect the medium’s pH, influencing the coloration of fungal structures. The fungistatic effect is primarily attributed to polyphenols such as catechin, epicatechin, and chlorogenic acid. These compounds contain hydroxyl groups that neutralize reactive oxygen species (ROS), preventing oxidative stress. Additionally, the phenolic ring provides conjugation and resonance properties, allowing electrons to move freely within the ring and conferring stability to its structure. This facilitates interaction with the cell membranes of *V. theobromae*, leading to permeabilization and release of vital cellular components. Consequently, enzymatic activity is altered, affecting protein and lipid structures. Furthermore, this process restricts oxygen consumption, disrupting the respiratory chain [37] and ultimately enhancing fungal growth inhibition.

The maximum inhibition reported for *V. theobromae* was 61.60% at 1000 ppm, a value exceeding 50%, which is considered acceptable for classifying an extract as efficient for controlling a fungal pathogen [38]. However, for *C. musae*, the maximum inhibition was 35.67% at 1000 ppm, and for *A. niger*, it was 27.59% at the maximum concentration of 2200 ppm. These values are lower than 50% because these fungi are more resistant compared to *V. theobromae*, requiring a higher extract concentration.

Differences in *V. theobromae* growth were observed from day 1, but they did not become significant until day 8, when differences between the effects of the applied concentrations increased. On the last day, a marked and significant difference in fungal inhibition was observed. In a study by [39], lemon and grapefruit extracts exhibited inhibition percentages of 54% and 48.85% at a maximum concentration of 500 ppm, which are lower values than those reported in this study. Initially, *V. theobromae* colonies tend to be white and gradually change to dark gray or black as they grow. However, at extract concentrations ranging from 600 ppm to 1000 ppm, the colonies remained white during the 16-day incubation period. This color preservation may be primarily due to genetic changes in the fungus, including mutations in the gene responsible for pigmentation, altering its normal production and expression. Additionally, in the presence of inhibitory compounds, the fungus can activate physiological defense structures, such as resistance forms characterized by compact mycelium composed of extensive hyphae. These structures enhance the absorption of active principles in the PDA, resulting in a greater effect on the pathogen and reducing its growth rate [40].

During the growth of *C. musae*, significant differences were observed starting from day 3 across all five concentrations, except for 200 ppm and the control. By the final day (day 16), a distinct difference from the control was evident, with moderate to low inhibition, reaching a maximum value of 35.67%. In a previous study, applying a 50% ethanolic extract of plantain (*llantén*) resulted in a higher inhibition of 68%. However, it is important to note that those extracts were not concentrated. The increased inhibition observed in the previous study can be attributed to ethanol, which interferes with the fungal cell membrane and energy metabolism, as reported by [41].

In another study, lemon and grapefruit extracts exhibited inhibitions of 30.38% and 22.6%, respectively. These values are similar to our findings, as both lemon and grapefruit contain flavonoids such as hesperidin, rutin, and quercetin. Additionally, a study made by [42] achieved 100% inhibition using an aqueous extract of *Nanshancha* seeds (from Southern China) at 5000 ppm. This high efficiency is attributed to the larger volume of extract used and the primary component—tea saponin—which possesses antimicrobial and antifungal properties due to its chemical structure (steroidal glycoside). Similarly, a 100% fungicidal effect was observed using 1% and 1.5% chitosan [43]. These results highlight that the polyphenols present in the avocado peel are not as effective against *C. musae*. This reduced efficacy may be due to the fungus developing greater resistance, possibly influenced by genetic or physiological factors, allowing it to tolerate the effects of the extract. Consequently, higher extract concentrations would be necessary to achieve inhibition beyond 50%.

While visual morphology did not exhibit significant changes in fungal development, inhibition of spore germination was evident. At 1000 ppm, the mycelium appeared less dense and flattened, further supporting the inhibition of spore germination, as reported by [19].

For the antifungal analysis of *A. niger*, the extract concentrations were doubled. Preliminary tests did not yield inhibition greater than 10%. However, the maximum inhibition achieved was 27.59%. On the first day of *A. niger* growth, no significant differences were identified between concentrations ranging from 1300 to 2200 ppm. By the sixth day, marked differences were observed, except at 1900 and 2200 ppm. This trend indicates that as the extract concentration increases, *A. niger* growth decreases, resulting in increased fungal inhibition.

In a study by [44], an inhibition percentage of 11.82% and 14.35% against *A. niger* was reported, which are lower values than those obtained in our study. Specifically, we achieved inhibitions of 16.61% and 27.59% at 1900 ppm and 2200 ppm on day 10. In a similar study conducted by [45], using 95% ethanol and maceration, a maximum inhibition of 5.67% was observed at 800 ppm when applying eucalyptus extract. This lower inhibition can be attributed to the use of lower extract concentrations and the specific chemical structure of the compounds obtained (such as eucalyptol).

Values below 50% are considered low inhibition. The adaptive capacity of *A. niger* to different stress conditions may explain this result. As an opportunistic pathogen found in the environment, *A. niger* can develop resistance and rapid proliferation [46]. Additionally, the possible generation of antioxidant enzymes such as superoxide dismutase and catalase counteract the effect of polyphenols on the fungal cell membranes, as reported by [47].

The effects of the extract on the fungus were evident in the physiology of *A. niger*. Until the fifth day, no spore formation was observed due to biochemical factors generated by the extract’s polyphenols, inducing a state of dormancy or rest. However, starting from the sixth day, spore generation was reactivated [48]. Despite clear mycelial growth at all extract concentrations, *A. niger* has a thicker cell wall (a physical barrier) that limits polyphenol entry and reduces its effectiveness. Nevertheless, spore inhibition was significant compared to the control plate. The use of parafilm to seal the plates reduced oxygen availability, affecting *A. niger*’s respiration and creating hypoxic conditions. Additionally, the mycelial growth appeared as a dense mass with branching chains of conidia, covering the poisoned PDA surface extensively. Despite using a higher extract concentration, the polyphenols’ effect was not efficient.

## 5. Conclusions

The utilization of avocado peel as a source of antifungal extracts holds great promise from both economic and environmental perspectives. Obtaining these extracts using unconventional methods, such as ultrasound-assisted extraction, coupled with optimal solvent concentration and appropriate extraction time, led to enhanced compound release and a high concentration of phenolic compounds, thus preventing their degradation. Our study focused on assessing the antioxidant capacity achieved through various extraction treatments. We evaluated two antioxidant analysis methods (DPPH and ORAC) and found that no prooxidant substances were formed. Notably, we can value the antioxidant potential of epicatechin, catechin, and chlorogenic acid—detected due to their molecular structure with aromatic rings and hydroxyl groups—that resulted in fungistatic effects, altering the growth and development of pathogenic organisms without complete inhibition. Additionally, we investigated physiological changes, including spore inhibition, alterations in mycelial density, and variations in normal fungal coloration. The best avocado peel extract (from the Hass variety) exhibited significant inhibition against banana-isolated fungal pathogens. Our findings revealed a direct implication of total phenolic compound concentration and high antioxidant activity against *Verticillium theobromae*, surpassing 50% inhibition until the last day of radial fungal growth. This sensitivity of the fungus to polyphenols led to reduced growth and structural modifications. The most effective extraction treatment involved 80% ethanol and 1 h and 30 min of extraction time, resulting in a phenolic content of 138.97 mg GAE/g dry sample—remarkably high compared to other studies in the literature. Additionally, considerable antioxidant capacity values were obtained due to the direct proportional relationship, allowing for radical neutralization in each assay. However, despite the significant antioxidant compounds in the extract, efficient antifungal activity was not guaranteed, especially since the mechanism of action of these antioxidants in avocado peel extract is not directly related to specific antifungal mechanisms. Consequently, inhibition remained below 50% for *Colletotrichum musae* and *Aspergillus niger*. Although these fungi were highly resistant to hydroethanolic avocado peel extracts, slight changes in growth diameter were observed, along with an impact on spore generation. This suggests adverse effects on fungal structure. Therefore, reusing agroindustrial waste contributes to waste reduction, reduces reliance on synthetic fungicides, and promotes a more sustainable approach in agriculture.

## Figures and Tables

**Table 1:** Total soluble polyphenol content in different extraction treatments.

	TPC (mg GAE/g DW)
TI	138.47 ± 0.006 ^a^
TII	129.55 ± 0.010 ^b^
TIII	130.94 ± 0.003 ^b^
TIV	124.89 ± 0.013 ^c^

Legend: TI—ethanol 80%/time 1 h30; TII—ethanol 80%/time 2 h00; TIII—ethanol 96%/time 1 h30; TIV—ethanol 96%/time 2 h; TPC—total phenolic content. Values followed by different letters are significantly different among the analyzed treatments, while values followed by the same letter are not significant.

**Table 2:** HPLC data of avocado peel extracts.

	Average Concentration (mg/L)	Retention Time (min)
chlorogenic acid	74.91	14.640
catechin	266.09	14.851
epicatechin	544.252	15.520

**Table 3:** Antioxidant capacity (DPPH inhibition %, ORAC) during the different treatments of extraction.

	DPPH (µmol Trolox/g DW)/% Inhibition	ORAC (µmol Trolox/g DW)
TI	833.32 ± 0.0051 ^b^/84.97%	1889.1 ± 0.50 ^a^
TII	822.80 ± 0.0059 ^a^/83.87%	1869.8 ± 0.60 ^ab^
TIII	823.41 ± 0.0088 ^a^/83.94%	1881.5 ± 0.21 ^b^
TIV	824.33 ± 0.0079 ^a^/84.04%	1843.7 ± 0 ^b^

Legend: TI—ethanol 80%/time 1 h30; TII—ethanol 80%/ time 2 h; TIII—ethanol 96%/ time 1 h30; TIV—ethanol 96%/time 2 h00. The values followed by different letters are significantly different among the analyzed treatments, while the values followed by the same letter are not significant.

**Table 4:** Percentage of mycelial growth inhibition (%) of pathogens (A) and (B) isolated from avocado peel exposed to various concentrations of ethanol extract compared to control stored at ambient temperature (26 °C).

Concentrations $(gL^{-1}$)	*Verticillium theobromae* (Day 16)	*Colletotrichum musae* (Day 6)
1000	61.59 ^a^ (0.704)	35.67 ^a^ (0.610)
800	50.92 ^b^ (0.610)	33.54 ^b^ (0.704)
600	28.97 ^c^ (0.610)	22.56 ^c^ (0.704)
400	19.82 ^d^ (0.610)	9.45 ^d^ (0.610)
200	8.23 ^e^ (0.610)	5.49 ^e^ (1.220)
Control PDA	0.30 ^f^ (0.000)	0 ^f^ (0.000)

Legend: Different lowercase letters within the column represent a significant difference between different concentrations, respectively. Means are separated using the Duncan test (*p* = 0.05). Each data point represents the average of four replicates; the standard deviation (SD) is given in parentheses.

**Table 5:** Percentage of mycelial growth inhibition (%) of the pathogen (C) isolated from avocado peel exposed to various concentrations of ethanol extract compared to control stored at ambient temperature (26 °C).

Concentrations ($gL^{-1}$)	*Aspergillus niger* (Day 16)
2200	27.59 ^a^ (0.584)
1900	16.62 ^b^ (0.584)
1600	11.43 ^c^ (0.584)
1300	5.95 ^d^ (0.584)
1000	3.05 ^e^ (0.584)
Control PDA	0 ^f^ (0.000)

Legend: Different lowercase letters within the column represent a significant difference between different concentrations, respectively. Means are separated using the Duncan test (*p* = 0.05). Each data point represents the average of four replicates; the standard deviation (SD) is given in parentheses.

## Data Availability

The original contributions presented in the study are included in the article, further inquiries can be directed to the corresponding author due to privacy.
